# Understanding the perspectives and needs towards midwifery care of women with an eating disorder history during pregnancy and postpartum: A qualitative study

**DOI:** 10.18332/ejm/216376

**Published:** 2026-02-28

**Authors:** Pauline Schmid, Annica F. Dörsam, Kathrin Schag, Katrin E. Giel, Claudia F. Plappert, Jana Katharina Throm

**Affiliations:** 1 Department of Internal Medicine VI-Psychosomatic Medicine and Psychotherapy, University Hospital Tübingen, Tübingen, Germany; 2 Centre of Excellence for Eating Disorders Tübingen (KOMET), University Hospital Tübingen, Tübingen, Germany; 3 German Center for Mental Health (DZPG), Berlin, Germany; 4 Department of Midwifery Science, Institute for Health Sciences, University Hospital Tübingen, Tübingen, Germany

**Keywords:** pregnancy, eating disorders, postpartum, midwifery care, womens needs

## Abstract

**INTRODUCTION:**

Eating disorders (EDs) can affect pregnancy outcomes in multiple ways. This study aims to contribute to the literature about whether and how midwives can support women with EDs in pregnancy and postpartum, and what they should consider when caring for affected women.

**METHODS:**

Mothers and becoming mothers who had experienced midwifery care and had a confirmed ED were invited to participate in this qualitative study. An interview guide was created that covered the needs of women with ED history in midwifery care, the influence of midwifery care on the ED, and support options provided by the midwife. In 2023, in Germany, five semi-structured interviews were conducted. The interviews were analyzed using Mayring's qualitative content analysis.

**RESULTS:**

Seven main themes emerged from the thematic analysis: ‘Influence of midwifery care’ on the ED, ‘Addressing ED’, ‘Nutrition and weight of the woman’, ‘Nutrition and weight of the child’, ‘Caring for the woman’, ‘Negative actions’, and ‘Midwife characteristics and services’. Women with ED history want midwives to ask them about EDs and remain alert to behaviors that may indicate ED. The topics of nutrition and weight changes, for mother and child, were highly relevant. Midwives should be aware of potential triggers and should continually work to strengthen the woman's body image and their self-efficacy.

**CONCLUSIONS:**

Women with a history of ED place special requirements on midwives to ensure a positive impact of midwife care. The categories identified in this study can serve as a starting point to further investigate the needs of affected women to provide adequate support to women with an ED history during midwifery care.

## INTRODUCTION

Eating disorders (EDs) are severe and complex mental disorders that predominantly affect women. The prevalence of EDs during pregnancy varies considerably in the literature between 1.0% and 8.4%, depending on the diagnostic criteria and the country^1-5^. Given this prevalence, it is likely that midwives will be caring for women with a history of ED over time.

A pregnancy and postpartum period can affect a pre-existing, even remitted ED for the better or worse, or might trigger the onset of an ED^2,6-12^. Some of these aspects were recently described by Sommerfeldt et al.^13^ as five possible trajectories perceived by women during pregnancy and early motherhood. The prevalence of an ED increases the risk of complications during pregnancy, labor and birth, such as an increased risk of premature birth, postpartum hemorrhage or abnormal weight development of the foetus^6,10,14-18^. Maternal EDs might also influence the child’s development beyond pregnancy, e.g. children are more likely to have emotional, behavioral or neurocognitive problems, lower weight and height development and feeding problems^19-23^.

Women with ED face many challenges, which might be intensified in the prospect of a pregnancy. In their qualitative work, Claydon et al.^24^ identified six themes which were prominent for pregnant women with EDs: control, disclosure to others, battle between mothering & ED, fear of intergenerational transmission of the disease, weight and body image concerns, and coping strategies. Affected women often also struggle with role adjustment and negative self-image^25^, and anxiety and depression are often additional aggravating factors^2,6,9,14,15,24^. Simultaneously, pregnancy is considered a particularly ‘teachable moment’, when pregnant women are especially receptive to health promotion advice^26^. Especially for primiparous women, pregnancy is a promising time for interventions, as the desire to protect the unborn child can be a strong motivation to refrain from ED behaviors, and the acceptance of one’s own body is often increased in the second half of pregnancy^8,25,27^. It is therefore crucial for midwives to understand the challenges of midwifery care for women with an ED history to adequately support them. As midwives have low-threshold access to women from all health and social backgrounds, they could make an important contribution to recognizing new or exacerbating EDs during pregnancy and may be able to initiate appropriate interventions to counteract the possible consequences of EDs for both mother and child.

Women in the German healthcare system are eligible for regular prenatal care by an obstetrician and midwife, financed by health insurance, and for postpartum midwifery care until the end of breastfeeding, usually by home visits. These outpatient midwives usually work as freelancers. In most cases, pregnant women only take advantage of medical prenatal care, as the entitlement to midwifery prenatal care is not well known. In addition, many midwives do not offer prenatal care because outpatient, freelance midwifery has been facing structural challenges. Some midwives offer at least one prenatal appointment to take her medical history, but often midwives make their first visit postpartum. Despite a shortage of midwives in many regions, too, most women use postpartum midwifery care; however, some women get only a few or no midwife appointments.

This study aims to contribute to the literature about whether and how midwives can support women with EDs in pregnancy and postpartum, and what they should consider when caring for affected women. This study addresses three questions: 1) ‘What special needs do women with a history of ED have in midwifery care, and what do midwives need to pay particular attention to in their care?’; 2) ‘Does midwifery care have an influence on ED?’; and 3) ‘What can midwives do to support women with a history of ED to cope with their ED during labor and postpartum?’.

## METHODS

### Study design

A qualitative approach was chosen. Semi-structured interviews were conducted with women who had a history of ED and received midwifery care.

The interviews took place at the participants’ homes in 2023 around Tübingen, Germany. All interviews were conducted by the first author of this study, a student of midwifery care with no personal connections to the participants. A total of five qualitative interviews with women with a history of ED during pregnancy were conducted.

### Recruitment process

The following inclusion criteria were defined: majority and capacity to consent; current or previous pregnancy <3 years; professional diagnosis of an ED at the time of pregnancy or in the past; and sufficient knowledge of the German language. Exclusion criteria comprised serious complications during pregnancy, severe medical conditions, or the presence of a serious illness in the child. The study was conducted at the Department of Psychosomatic Medicine and Psychotherapy at the University Hospital Tübingen as a sub-study of the EMKIE family cohort study^12,18,19,28^. The recruitment process for the EMKIE study has been described by Doersam et al.^28^. Participants in the EMKIE study, who met the inclusion criteria, were asked to participate in this sub-study in descending order of temporal proximity to delivery, until the desired number of five participants was reached.

### Sample characteristics

Five mothers with an ED history who had experienced midwifery care were interviewed. The participants were aged 28–34 years (mean age: 30.4 ± 2.9 years) and had one or two children aged between a few weeks and five years (mean age: 1.8 ± 1.8 years). All participants spoke German as their mother tongue, lived in partnership with the fathers of their children, and were of high socioeconomic status. Four participants had a history of anorexia nervosa (AN), one had a history of bulimia nervosa (BN), and one participant had a comorbid borderline diagnosis. The self-reported duration of ED history among participants ranged from 12 to 20 years. All participants reported being in remission at the time of the interview, which in this context means that they had no severe symptoms and were not undergoing treatment back then. However, this information was self-reported, and there were no data about the duration of the remission. Three women received prenatal care, or at least one prenatal appointment with a midwife during one of their pregnancies, and all women received postpartum midwifery care.

### Interview process and guide

An interview guide was created using the collect-check-sort-subsume method^29^. The interview guide contained the following four overarching questions: ‘With regard to your history of ED, which specific needs in midwifery care do you mind?’, ‘Did you feel that midwifery care had an impact on your ED, if so, positive or negative?’, ‘What can midwives do to support people with a history of ED to cope well with it during pregnancy and the postpartum period?’, and ‘What can midwives learn from your experience with midwifery care in the context of ED?’. These questions were followed up with other questions designed to keep the conversation going and lead the interview to study-relevant topics, such as: ‘How can midwives address special needs?’, ‘What did you expect from midwifery care in regard to your ED?’, ‘What specific counselling content is important for you regarding your ED?’, ‘Which behavior and treatment by your midwife had a positive or negative effect on your ED?’, ‘Which aspects of care are lacking?’, and ‘Which are the advantages of midwifery care for EDs and which influence does it have?’. To increase the openness and objectivity of the questions, the interview guide was checked by a second researcher.

### Data analysis

Transcription of the interviews was carried out according to Dresing and Pehl^30^. Data analysis was conducted by the first author using Mayring’s qualitative content analysis^31^ with inductive category development during an iterative coding process. The transcribed interviews were first divided into coding units. Coding units relevant to answering the research questions were paraphrased and labelled with keywords. These keywords represent the subcategories of the category system. The subcategories were grouped into main categories. After 40% of the material had been assigned, the created categories were sorted and grouped into a structured category system. In the next step, the missing coding units were assigned to this category system.

### Ethics and transparency

The Ethics Committee of the Medical Faculty of the University Hospital Tübingen approved the conduct of the study (493/2023BO2). All participants provided written informed consent. In order to ensure transparency regarding the quality of this study, the Standards for Reporting Qualitative Research Checklist (SRQR Checklist) was used and is attached in the Supplementary file.

## RESULTS

**Figure 1:**
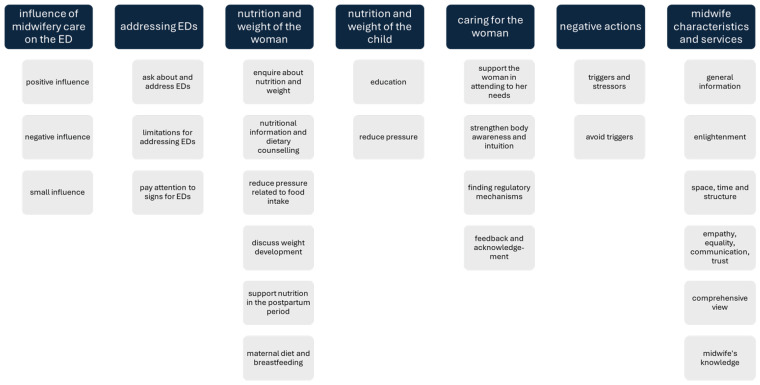
Category system. The category system was generated inductively from the interviews conducted. The interviews took place in Germany in 2023. Five women who had suffered from ED, had been pregnant at least once, and had received pre- or post-natal care from midwives, were interviewed. To generate the category system, the interviews were divided into text sections and tagged with keywords. These keywords form the subcategories of the category system. The subcategories were grouped into the seven main categories

The developed category system consists of seven main categories, each containing two to six subcategories (Figure 1).

### Influence of midwifery care on the ED

Three of the five women described an overall positive influence of midwifery care on their ED, while one participant experienced a negative influence of midwifery care on her ED, as the midwife made judgmental comments about the participant’s breasts. One participant reported that the midwifery care had only a small influence on her ED, as her midwife did not address EDs during her care. Further, she stated that most women meet their midwife only shortly before giving birth because of a shortage of midwives in Germany:

*‘Definitely positive for me, because [the midwife] simply encourages you from the first second, hey, your body can do it, listen to your body, your body is signaling what it needs …’* (Interview 5)

*‘I had the feeling that it was a bit negative because of the comment that my breasts were very small, for example. … the sensitivity was missing at that moment.’* (Interview 3)

### Addressing EDs

Except for one participant, the respondents were certain that it is acceptable, good, or necessary for the midwives to ask about and address EDs:

*‘I don’t know if I have hoped for it, but it would have been absolutely fine for me to talk about [the ED].’* (Interview 1)

The respondents mentioned limitations for asking about EDs and addressing EDs, including a long-time remission of the ED, fear of stigmatization, the need for an underlying relationship of trust to open, as well as the difficulty for the affected person in recognizing and naming their own need for help. The participants noted that midwives themselves might encounter barriers, including a general taboo around the topic and a lack of time. They described that the midwife should pay attention to signs for EDs, as affected women often do not talk about their ED directly. These signs include symptoms or concerns associated with EDs:

*‘… of course I didn’t tell her “Hey, … I had an eating disorder”, but I did ask her questions because I was often insecure because I had gained so little weight, … that my stomach was so small …’* (Interview 3)

### Nutrition and weight of the woman

Three participants expressed the wish that the midwife would enquire about nutrition and weight and evaluate together whether these aspects are sufficient and adequate. One participant described that she would have appreciated being asked by the midwife about her experience with weight gain:

*‘[the midwife] could have asked “Ok, you’re gaining so little weight now, so what’s your diet like”, so that you say what you eat, how much you eat, …’* (Interview 3)

The desire for nutritional information and dietary counselling from the midwife during pregnancy and in the postpartum period was articulated by four participants, as this would have or actually had given them reassurance. The women reported that advice on the importance of a balanced and adequate diet during pregnancy and recovery during the postpartum period was very beneficial. One participant also mentioned how comforting it was for her to disclose to the midwife when she was worried about overeating. Furthermore, midwives were seen as role models on the topic of nutrition:

*‘I would have liked a bit more information about nutrition in general, because I think that would have given me more assurance.’* (Interview 3)

According to one participant’s statements, the midwife was able to reduce pressure related to food intake by placing nutrition in relation to other needs of the mother and child. One participant described that it helped her that the midwife did not give information on nutrition in a lecturing and pressuring manner, while another participant had the opinion that individuals with ED need clear statements from the midwife, including the possible consequences of inadequate nutrition:

*‘… maybe it’s good for the mental health to hear that if you eat unhealthily for two or three days … I hope it’s not bad for the baby …’* (Interview 1)

The participants also expressed a desire to discuss weight development with the midwife. This included reassurance regarding weight changes during pregnancy and postpartum, as well as education on the connection between nutrition, weight development, and fetal development during pregnancy.

It was described as beneficial when the midwife supported nutrition in the postpartum period by providing concrete suggestions on how to simplify food supply, such as involving relatives. The participants also wished for information about the physiological demands of the body during the postpartum period to understand the importance of adequate nutrition.

Another requested aspect regarding maternal nutrition was the influence of maternal diet on breastfeeding, highlighting that breastfeeding is very demanding on the body and therefore the importance of sufficient nutritional supply:

*‘The feedback “You’re the mom of a little miracle, your body has managed that, and your body is good … but your body also needs a lot of food now.” ’* (Interview 4)

### Nutrition and weight of the child

The women expressed a desire for education regarding eating behaviors and the weight of their child from the midwife. This included information on what ‘normal’ eating behavior can look like for a child. Some judgmental comments regarding the child’s weight were perceived as very disturbing. One participant described a midwife-led breastfeeding café as highly beneficial, as it allowed mothers to exchange information about their children’s nutrition. It was difficult for two women when the midwife no longer visited the families at the time of complementary food introduction, and therefore, no education was provided on this topic:

*‘But maybe it would have been nice to hear for a person who has a disturbed relationship with food … what a normal relationship with food can look like for a baby.’* (Interview 1)

The midwife can reduce pressure related to nutritional concerns about the child by providing some concrete guidance which emphasizes that: a child’s nutrition is not the only important factor for the well-being of both mother and child; each family has its own optimal solution for child nutrition; it is not always necessary to choose what appears to be the healthiest option; and a child will receive adequate nutrient intake if a few key aspects are considered:

*‘Both during pregnancy and after birth, it was always important for me to hear “the whole package is important”. I always focused a lot of my worries and thoughts on the topic of nutrition, how I was feeding myself during pregnancy, and how I feed my baby after the birth. And I forgot about the whole package.’* (Interview 1)

### Caring for the woman

Participants found it helpful when the midwife supported the woman in attending to her needs by mirroring the woman’s feelings, asking about her requirements, and reminding her of her own needs when she was no longer aware of them:

*‘… and to have an empathic midwife who says “hey, I can see you’re not feeling well at the moment, what can I do for you?”, as an offer, just as an offer …’* (Interview 4)

Midwives can strengthen body awareness and intuition through various approaches, including prenatal yoga, postpartum exercises, reinforcing the ‘maternal intuition’, encouraging the perception of hunger and satiety, through prenatal care by the midwife in comparison to only obstetric care and the recurring statement ‘your body can do it, trust your body, it signals its needs’ throughout the entire period of care:

*‘In my first pregnancy, when I went to the obstetrician, I always felt a bit misplaced … Midwife-led [prenatal care]*

*… it’s a completely different body feeling …’* (Interview 5)

The participants described that the midwife should ask the woman about existing harmful behavioral patterns, signal that she recognizes the woman’s distress, and assist the woman in finding regulatory mechanisms that are compatible with pregnancy and the postpartum period:

*‘… I used to compensate a lot through sport, my emotions, and since the first pregnancy, I haven’t had that tool because you can no longer go running to the same extent, … you need a tool to coregulate yourself a bit, and the midwife can simply give you advice about that …’* (Interview 5)

Feedback and acknowledgement by the midwife for the achievement of a sufficient diet were also perceived as helpful.

### Negative actions

Each participant described triggers and stressors related to the ED that could occur or occurred during midwifery care. Any comment about the baby’s milk intake and weight, inconsistent recommendations from the midwife about the baby’s nutrition, the maternal weighing during pregnancy checkups, any comments about breast size, being requested to eat more, being taught what the body cannot supply on its own and therefore needs to be substituted, and the substitution of iodine tablets, may all constitute closely monitoring nutritional values and serve as significant triggers:

*‘I always experienced weighing quite horrible, even at the obstetrician’s during both pregnancies, always this gaining of weight, that the scales showed more weight, I found that horrible.’* (Interview 2)

*‘But if you’re already struggling a bit with your body as a woman, and then you hear that one hormone is missing, and something else is missing as well, and you have to substitute it, then I asked myself if my body can do something on its own.’* (Interview 5)

One participant mentioned that the midwife can avoid triggers by having the empathy to know the right moment to bring up the topics of nutrition and weight, providing information material so that every woman can manage the information requirements herself, and using sensitive language.

### Midwife characteristics and services

Participants appreciated when the midwife provided general information on nutrition to all women, for example, in antenatal courses. Creating opportunities for women to talk to each other and midwives about the nutrition of the baby and other topics, such as joint acupuncture sessions or breastfeeding cafés, was considered helpful in managing EDs.

The participants described the wish to receive enlightenment from the midwife about the physical changes, the time the body needs after birth to return to a similar shape as before pregnancy, the softening effect of breastfeeding hormones on the body, and the consequences of excessive exercise on the postpartum body.

The aspects of space, time, and structure that the midwife incorporated into her appointments were highly prominent during the interviews. Participants described that the environment during midwifery appointments was more suitable for opening than during the obstetric consultations and that the clear structure that the midwife had at her appointments gave the women a sense of control. Furthermore, the time and presence that the midwife offered facilitated the development of a trusting relationship:

*‘You actually need to have the midwife with you much more in advance [during pregnancy]. So that a basis of trust is developed. Because that doesn’t come from talking and ultrasounds.’* (Interview 5)

Three participants emphasized the importance of empathy, equality, communication, and trust in midwifery care, which served as the basis for a positive influence on the ED as well as the midwife’s comprehensive view of the woman’s entire situation:

*‘I think that’s what a lot of women need in this situation, that the midwife is not teaching and lecturing with authority … but simply takes you by the hand, picks you up where you are and communicates with you at eye level. Just like this: “I’m here for you, if there’s anything, get in touch, but I won’t impose it on you.” ’* (Interview 5)

The women felt that the midwife’s knowledge regarding certain aspects of EDs is fundamental. They specifically highlighted the importance of knowledge and evidence-based counselling on nutrition topics to mother and child, an understanding of the particularities and prevalence of various EDs, as well as a sensitivity towards these disorders to ask targeted questions and support improvements. It is crucial for the midwife to recognize that individuals with a history of EDs often have a strong fixation on food, and that disturbances of body image can remain a lifelong concern for those affected, even after they have recovered physically. Furthermore, there is a risk that affected individuals may revert to old behavioral patterns in response to acute dissatisfaction with their bodies:

*‘Simply that midwives are a bit sensitive to the fact … that many women may be affected …’* (Interview 3)

## DISCUSSION

In view of the different risks associated with an ED history for the physical and mental health of mother and child, it is clear how important it is for midwives to face the special needs of affected women in their care. Concrete recommendations for practical midwifery work can be deduced from the results of this study, which emerge from the seven thematic topics developed. Some of the recommendations correspond to existing literature, thereby increasing their relevance.

Midwives can have a positive impact on the prepartum and postpartum care of women with an ED history, provided that they comply with certain requirements. Primarily, midwives need an awareness of EDs. This supports previous research emphasizing the importance for healthcare professionals working with pregnant women to understand EDs well and to recognize them during pregnancy to intervene effectively^19,32,33^. It is additionally important for midwives to know the limits of their profession and work interprofessionally^34^. Furthermore, this study also shows that comprehensive, continuous midwifery care based on a trusting relationship is crucial to achieving positive effects on ED. This result is supported by the common fact that continuous midwife care is a major health-promoting factor in general^33,35,36^. Simultaneously, midwives should keep in mind that they can unintentionally exert a negative influence on the ED by addressing the woman’s triggers. So, midwives should be aware of potential triggers, ask the woman about them and help making them more manageable for the woman if they are unavoidable. In her practical work, the midwife will face many situations that require her to keep a balance between medically necessary examinations and advice on the one hand and a sensitive approach to the woman’s triggers on the other. In addition to the situations mentioned by the participants, other triggering situations could be the screening for gestational diabetes, any necessary dietary rules for gestational diabetes, palpating the uterus, or the feeling that the body is failing if medical interventions become necessary.

The results further emphasize that a comprehensive anamnesis is essential in midwifery care. In accordance with the work of Pettersson et al.^33^, our results show that midwives should specifically ask women about ED history and current eating behavior. ‘Disclosure to others’ is a prominent topic for pregnant women with ED^24^ underlining that the midwife must be particularly attentive about certain statements or behaviors exhibited by the woman which could possibly indicate an ED. Other studies mentioned that the disciplines involved in the care of affected women during pregnancy, such as obstetricians, psychotherapists, and midwives, should ensure that maternal mental health, ED pathology, adaptation to motherhood, and maternal nutrition are regularly monitored and help is offered^6,11,15,19,32^. Ecob et al.^34^ also highlighted the importance of early detection, screening, and monitoring of ED during pregnancy and postpartum. These aspects must also be considered by midwives, but were not mentioned by the participants in our study.

Our findings showed that issues related to personal nutrition, weight changes, and the nutrition of the child are important for many women with a history of ED in midwifery care. It is therefore important for midwives to sensitively address the physical changes and weight changes that occur during pregnancy and postpartum, explain their necessity, and discuss with the woman how she is coping with them. The midwife should also inquire about the woman’s nutrition, collaboratively assess whether her dietary intake is adequate, advise the woman on nutrition, and maintain a balance between non-instructive communication at eye level and pointing out the possible consequences of inadequate nutrition. Particularly in the postpartum period, when the parents’ focus is usually mainly on the baby, the midwife should support the woman’s nutrition with practical tips and emphasize the need for adequate nutrition during breastfeeding. This should address more than the general maternity care topics, such as the supplementation of folic acid and iodine. On the other hand, some of the participants thought that the midwife can relieve the woman by reducing pressure related to her diet. This ambivalence could be caused by different trajectories of the EDs women experience during pregnancy and postpartum, and therefore their different needs depending on the type and trajectory of their ED^13^. In clinical practice, the difficulty is balancing between nutritive advice that complies with medical requirements and not drawing the focus too much on the diet, which is potentially associated with triggering the pathomechanism of EDs.

When addressing the baby’s weight and nutrition, midwives should be very sensitive. They should discuss the baby’s diet and show the mother what an adequate diet for the baby could look like. This might provide the women with a sense of reassurance and alleviate their concerns regarding the possibility of transgenerational transmission of the disease, which was mentioned as a prominent issue in pregnant women with ED history^24^. However, advising women about both nutrition and weight development, even for their child, without boosting their pathological focus on this topic, remains difficult.

Regardless of a woman’s medical conditions, many midwives consider it part of their professional identity to empower women and strengthen their body awareness. Based on these studies’ results, strengthening the woman’s body awareness, intuition, and trust in her body is even more important in the context of an ED history. This aligns with existing literature, which indicates that treatment for EDs surrounding pregnancy should particularly address body image disturbances and the self-critical maternal image^35^. A sensitive perception and reflection of the woman’s well-being enable her to recognize her own situation and needs. The midwife can work collaboratively with the woman to identify harmful behavioral patterns and find harmless regulatory mechanisms called ‘self-help strategies’ by Pettersson et al.^33^. As examples, they listed distraction, an additional meal per day, and eating new foods or motivating themselves by taking the perspectives of the child or partner^33^. To strengthen the woman, it can be useful to offer prenatal care provided not only by the obstetrician but also by the midwife, to offer adapted pre- and postpartum exercises to assist the woman to gain a better feeling for and control over her body, and to empower the woman’s self-efficacy in her role as a mother. As the body image might be negatively affected by the ED, midwives can try to improve the women’s acceptance of the physiological body changes during pregnancy and the postpartum period using well-teachable procedures. For example, instructing women to palpate their own bellies and observe the growth of the uterus as well as its postpartum regression, helps establish a direct connection to the natural body mechanisms, while demonstrating colostrum extraction could be a good way to encourage women to interact positively with their bodies.

To implement the described recommendations in the German healthcare system, primarily, each pregnant woman should have the opportunity for prenatal and postnatal midwifery care, which correlates inter alia with the capacity of freelancing midwives. At the time of the interviews, midwives in Germany earned very little for postpartum care, so midwives had to keep visits short to work profitable. It is therefore encouraging that a new regulation was introduced recently, allowing midwives to account for longer appointments, enabling them to address issues such as EDs. Obstetricians as primary contact for pregnant women should be aware that women with a positive ED history can benefit in particular from shared prenatal care by midwives and obstetricians, recommending this to their patients. Psychology professionals can also act as mediators and suggest midwifery care to pregnant women. Midwives should also try to stay up to date with the latest research about this topic. Furthermore, to enable midwives to be trained, EDs should be given sufficient consideration in midwifery degree courses. Both allow midwives to positively utilize the teachable moment of pregnancy and motherhood for women with ED, thereby helping women in the long-term.

### Strengths and limitations

This study offers an understanding of the expectations of women with ED history towards midwifery care regarding their disease, which represents a new contribution to the field. The results are directly relevant to the daily work of midwives in the prenatal and postpartum care of affected women and should be considered by midwives, especially since many of the conclusions can be implemented in practical work with little effort. A woman-centered approach was chosen to make the needs of affected individuals more understandable for midwives. The standards for reporting qualitative research (SRQR) checklist was used to make the methodological approach more comprehensible and verifiable for the reader.

The five interviews conducted provide a good basis for exploring the relevant aspects, but further interviews with larger samples are necessary to map the needs more comprehensively. The self-developed interview guide was not pre-validated in a test run, and the category system was developed only by the first author. All participants self reported that they had remitted from their eating disorder before or during pregnancy, limiting the applicability of the findings to other samples. Additionally, all participants had a high socioeconomic status, causing a bias versus the actual collective. Furthermore, no distinction was made between participants with anorexia nervosa and bulimia nervosa, so that conclusions were drawn for EDs in general; however, some may only apply to a specific ED. While an ED diagnosis was granted due to subsampling participants from the EMKIE study, the history of their illness in this study is only based on the information provided by the women themselves during the interview. This means that only limited and insufficiently validated information is available for classifying the results.

### Future research

Further studies are necessary to capture diverse perspectives from individuals in various life situations and with different EDs at different stages of the disease. The herein presented categories can serve as a starting point to deepen the knowledge about the requirements of affected women and to further explore the conclusions from this study, also with quantitative research. The partner appears to be an important resource for women, as mentioned by some participants in this study. How midwives can effectively involve the partner as a resource for women and how they can make the best possible use of these positive effects seems to be an interesting aspect, which was not a focus of this work.

## CONCLUSIONS

Women with a history of EDs place special requirements on midwives to ensure a positive impact of midwifery care. Therefore, a variety of key advice needs to be considered and individually adapted to the women affected. Those affected would like the midwife to ask about and address the ED. Important topics in the care are maternal and infant nutrition and weight changes. Midwives should strengthen the body image of those affected, help them to find suitable regulatory mechanisms, and help them to be aware of their needs. Midwives should be sensitive to potentially triggering situations that may arise during midwifery care. Successful relationship building through sufficient time and respectful communication is a prerequisite for successful care. The findings of this study can be used as a basis for further research into the needs of women with a history of ED during midwifery care, in order to ensure appropriate support.

## Supplementary Material



## Data Availability

The data supporting this research are available from the authors on reasonable request.
